# Supplementary material for: Assessing the Physiochemical Parameters and Reduction Efficiency from Two Typical Wastewater Treatment Plants in the Vhembe District in South Africa
Source: Int J Environ Res Public Health. 2025 May 30;22(6):856. doi: 10.3390/ijerph22060856 (PMC12193668; doi:10.3390/ijerph22060856)
Supplement: Supplementary file 1 [file ijerph-22-00856-s001.zip › ijerph-3635069-supplementary.pdf]

S1: 1A and B

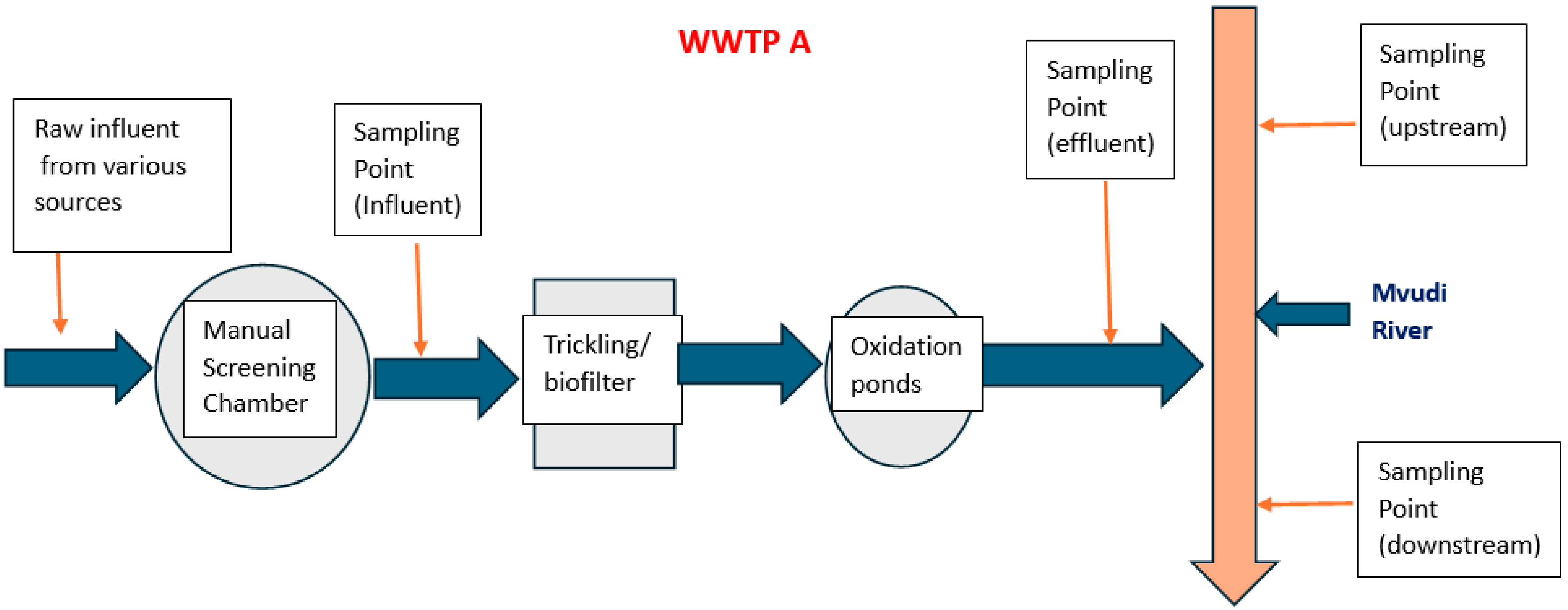

Supplementary material Figure S1: Schematic diagram of the technological sequence of WWTP A

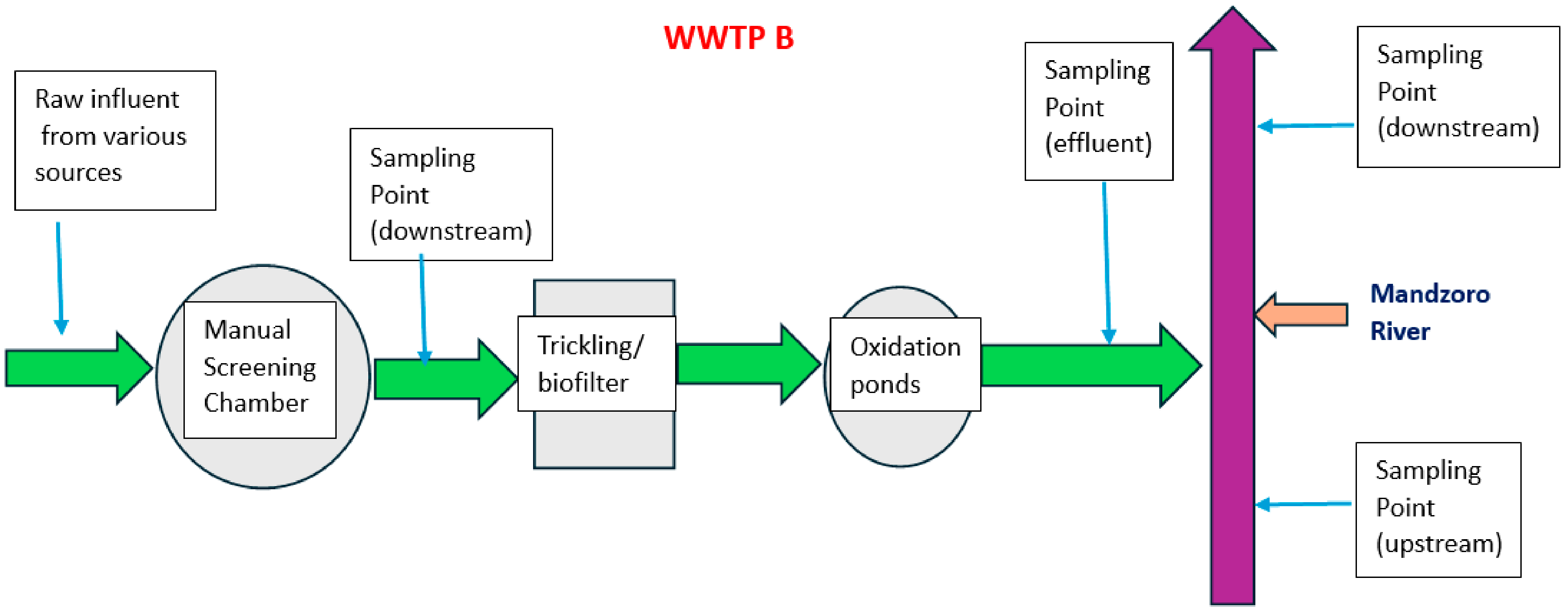

Supplementary material Figure S2: Schematic diagram of the technological sequence of WWTP B

File S1: **Physicochemical analysis protocols**

All equipment and meters for analysis of the parameters were calibrated and inspected according to the manufacturer's instructions (*www.hannainst.com)*. The physicochemical parameters that were analysed were temperature, pH, electrical conductivity (EC), total dissolved solids (TDS), dissolved oxygen (DO), chemical oxygen demand (COD), alkalinity, as well as the concentration of nitrate, nitrite, phosphate, sulphate, chloride, and ammonium, turbidity, and free chlorine. They were analysed using a Hanna HI98194 multi-parameter reader and an H1801 spectrophotometer iris Parameter, both manufactured by Hanna Instruments in the USA. In the laboratory, the turbidity analysis was also performed on-site using a Lovibond TB 211 turbidimeter (Lovibond. IR, Germany).

Temperature, pH, DO, EC and TDS

These were analysed using a Hanna HI98194 multi-parameter according to the manufacturer's instructions (*www.hannainst.com)* with the help of their respective individual probe after calibration to ºC for temperature, different buffers (4, 7 and 10) for pH, ppm or % saturation for DO, µS/cm or mS/cm and ppm or mg/L for TDS directly into the sample then read the output.

Turbidity

This is analysed by the Lovibond TB 211 turbidimeter which was first calibrated using formazin-based standards, typically with known turbidity values like <0.1, 20, 200, and 800 NTU. The samples were placed in the vial to the specified line with the water sample to be analyzed as the instrument measured the scattered light at 90°. The data obtained were interpreted as Nephelometric Turbidity Units (NTU).

Nitrite

The Nitrite HR method was selected according to the manufacturer's instructions (*www.hannainst.com)* where a cuvette was filled with 10 ml of unreacted sample, capped, and placed in the holder after calibration of the instrument. A sachet of Nitrite reagent was added to the cuvette and mixed thoroughly. The reaction mixture was left for 10 minutes and the concentration was determined using a H1801 spectrophotometer iris at a wavelength of 575 nm.

Nitrate

The Nitrate method was chosen following the guidelines of the Factory Methods procedure (*www.hannainst.com)*. A cuvette was filled with 10 mL of the sample, and a packet of Nitrate Reagent was added. Subsequently, the mixture was shaken vigorously for 10 seconds. The concentration of the nitrate-nitrogen (NO_3-N) mixture was analysed using a spectrophotometer at a wavelength of 525 nm.

Sulphate

The Sulphate method was implemented based on the Factory Methods procedure (*www.hannainst.com)*. A cuvette containing 10 mL of unreacted sample was capped and placed in the holder after calibration. One packet of Sulphate Reagent was added, and the cuvette was gently inverted for about 1 minute. The sulphate (SO_4) concentration in mg/L was then determined using a spectrophotometer at a wavelength of 466 nm.

Phosphate

The Phosphate method was implemented according to the Factory Methods procedure (www.hannainst.com). A cuvette containing 10 mL of unreacted sample was capped and placed in the holder after calibration. Ten drops of Phosphate High Range Reagent A and one packet of Phosphate High Range Reagent B were added, and the cuvette was gently shaken until the reagents were fully dissolved. After allowing a reaction time of 5 minutes, the presence of phosphate was confirmed by the appearance of a blue colour. The phosphate concentration in mg/L was then determined using a spectrophotometer at a wavelength of 525 nm.

Alkalinity

The Alkalinity method was performed according to Factory Methods directives (*www.hannainst.com)*. A cuvette containing 10 mL of unreacted sample was capped and inserted into the holder after calibration. The cuvette was removed, and 1 mL of Reagent was added with a syringe. The cuvette was inverted 5 times to mix. It was then reinserted into the holder, where the alkalinity results in mg/L of calcium carbonate ($CaCO_3$) determined using a spectrophotometer at a wavelength of 610 nm.

Chlorine

The Chlorine method was implemented following standard procedures (*www.hannainst.com)*. A cuvette containing 10 mL of unreacted sample was capped and calibrated before proceeding. For Free Chlorine, one packet of Free Chlorine Reagent was added, and the cuvette was gently shaken for about 20 seconds. After a reaction time of 1 minute, the chlorine (Cl_2) concentration in mg/L was determined using a spectrophotometer at a wavelength of 525 nm, with the intensity of pink colour indicating concentration levels. For Total Chlorine, a similar procedure was followed, including the addition of a Total Chlorine Reagent packet, gentle shaking for 20 seconds, and a reaction time of 2 and a half minutes before analysis at the same wavelength, with corresponding colour variations indicating concentration levels.

Chloride

The Chloride method was implemented based on the manufacturer's instructions (*www.hannainst.com)*. A cuvette containing 10 mL of the sample was capped and placed in the holder after calibration, while another cuvette with 10 mL of deionized water was prepared as a blank. Following this, 0.5 mL of Chloride Reagents A and B were added to both cuvettes and mixed gently by inverting for approximately 30 seconds. The chloride (Cl^{-}) concentration in mg/L was then determined using a spectrophotometer at a wavelength of 455 nm.

Chemical Oxygen Demand (COD)

Heating Procedure

The digestion reactor was preheated to 150 °C. Two millilitres of the sample were added to the specific vials containing the digestion solution (a mixture of potassium dichromate, mercury sulphate, and concentrated sulfuric acid) and thoroughly mixed. The vials were placed into the reactor for two hours at 150 °c. After two hours of digestion, the reaction was cooled to room temperature and then analysed using a spectrophotometer alongside the blank at a wavelength of 610 nm.

Ammonia

The Ammonia MR method was selected as per manufacturer instructions (*www.hannainst.com)*. 10 ml of the sample was added to a 16 mm vial, and then four drops of ammonia reagents A and B were added and thoroughly mixed to achieve a homogenous mixture. The ammonia nitrogen (NH_3-N) concentration in mg/L was then analysed using a spectrophotometer at a wavelength of 425 nm.
